# Endophytic *Colletotrichum* species from *Dendrobium* spp. in China and Northern Thailand

**DOI:** 10.3897/mycokeys.43.25081

**Published:** 2018-12-04

**Authors:** Xiaoya Ma, Sureeporn Nontachaiyapoom, Ruvishika S. Jayawardena, Kevin D. yde, Eleni Gentekaki, Sixuan Zhou, Yixin Qian, Tingchi Wen, Jichuan Kang

**Affiliations:** 1 Engineering and Research Center for Southwest Biopharmaceutical Resource of National Education Ministry of China, Guizhou University, Guiyang 550025, Guizhou Province, People’s Republic of China; 2 Center of Excellence in Fungal Research, Mae Fah Luang University, Chiang Rai 57100, Thailand; 3 School of Science, Mae Fah Luang University, Chiang Rai 57100, Thailand; 4 Guizhou institute of animal husbandry and veterinary, Guiyang, Guizhou province, 550005, People’s Republic of China

**Keywords:** *
Colletotrichum
*, *
Dendrobium
*, Endophytic fungi, multi-loci, new species

## Abstract

Species of *Colletotrichum* are commonly found in many plant hosts as pathogens, endophytes and occasionally saprobes. Twenty-two *Colletotrichum* strains were isolated from three *Dendrobium* species – *D.cariniferum*, *D.catenatum* and *D.harveyanum*, as well as three unidentified species. The taxa were identified using morphological characterisation and phylogenetic analyses of ITS, GAPDH, ACT and ß–tubulin sequence data. This is the first time to identify endophytic fungi from *Dendrobium* orchids using the above method. The known species, *Colletotrichumboninense*, *C.camelliae-japonicae*, *C.fructicola, C.jiangxiense* and *C.orchidophilum* were identified as fungal endophytes of *Dendrobium* spp., along with the new species, *C.cariniferi*, *C.chiangraiense*, *C.doitungense*, *C.parallelophorum* and *C.watphraense*, which are introduced in this paper. One strain is recorded as an unidentified species. Corn meal agar is recommended as a good sporulation medium for *Colletotrichum* species. This is the first report of fungal endophytes associated with *Dendrobiumcariniferum* and *D.harveyanum*. *Colletotrichumcamelliae-japonicae*, *C.jiangxiense*, and *C.orchidophilum* are new host records for Thailand.

## Introduction

*Colletotrichum* is the sole genus in family *Glomerellaceae* (Glomerellales) (Maharachchikumbura et al. 2015, 2016; Jayawardena et al. 2016b; Hongsanan et al. 2017). Presently, there are 193 accepted *Colletotrichum* species in eleven species complexes and 23 accepted singleton species (Hyde et al. 2014; Jayawardena et al. 2016a). *Colletotrichum* species has been listed as one of the top ten fungal pathogenic genera in molecular plant pathology based on scientific/economic importance (Dean et al. 2012). Anthracnose caused by *Colletotrichum* species can be a devastating disease in many economically important crops, including fruit crops, vegetables, cassava, sorghum, as well as ornamental plant such as orchids (Prusky and Plumbley 1992; Hyde et al. 2009a, b; Cannon et al. 2012; Dean et al. 2012; Jadrane et al. 2012; Jayawardena et al. 2016a; Diao et al. 2017). Many pathogenic *Colletotrichum* species that adopt biotrophic life strategies are present as symptomless endophytes in living plant tissues (Photita et al. 2004), although a large number of non-pathogenic species also occur as endophytes (e.g. Mendgen and Matthias 2002; Lu et al. 2004; Rojas et al. 2010; Cannon et al. 2012; Kleemann et al. 2012). Interestingly, experiments of Redman et al. (2001) showed that pathogenic *Colletotrichum* species could express mutualistic lifestyles in plants not known to be hosts and conferred disease resistance, drought tolerance, and/or growth enhancement to the host plants. Even though the diversity of *Colletotrichum* species associated with cultivated plant hosts have extensively been studied (Yang et al. 2009), a very limited number of studies has been conducted on *Colletotrichum* species from non-cultivated plants in natural and semi-natural habitats (Cannon et al. 2012).

*Dendrobium* SW. is the second largest genus in *Orchidaceae* (The Plant List 2013). Most *Dendrobium* species/hybrids are important ornamental/floricultural crops, but some species within this genus also possess medicinal values (Xu et al. 1995; Ng et al. 2012). Many *Dendrobium* orchids have been listed as Chinese medicinal herbs and are used for the treatments of atrophic gastritis, diabetes, faucitis, fever, red tongue, and/or thirsty (Ping et al. 2003; Bulpitt et al. 2007; Xing et al. 2011; Xia et al. 2012; Xu et al. 2014). Moreover, some *Dendrobium* orchids including *D.catenatum* Lindl. (widely known as *D.officinale* Kimura & Migo) have been listed as critically endangered species by the International Union for Conservation of Nature (IUCN) (www.iucnredlist.org). Due to their significance, *Dendrobium* orchids have been the subject of many studies including the diversity of endophytic fungi (Ma et al. 2015). However, only a limited number of studies on endophytic *Colletotrichum* in *Dendrobium* species have been reported and the number of *Dendrobium* species included in these studies are very few (Yuan et al. 2009; Yang et al. 2011; Mangunwardoyo and Suciatmih 2011; Chen et al. 2012; Noireung et al. 2012; Tao et al. 2013). In the present study, we investigated the diversity of endophytic *Colletotrichum* in five *Dendrobium* orchid species collected from a mountain (at an elevation of 1,300–1,400 m) close to the Thailand-Myanmar border and *D.catenatum* collected from Guizhou Province in China. A total of 22 endophytic *Colletotrichum* strains were isolated and identified based on both morphological and molecular characteristics. Five *Colletotrichum* strains, *C.cariniferi*, *C.chiangraiense*, *C.doitungense*, *C.parallelophorum* and *C.watphraense* are introduced as new species. The results of this study will contribute to the knowledge on diversity and phylogeny of *Colletotrichum*.

## Material and methods

### Sample collection

Healthy roots, stems and leaves of *D.cariniferum*, *D.harveyanum* and three unidentified *Dendrobium* taxa (referred to as *Dendrobium* sp. 1, 2 and 3) were collected from Wat Phra That Doi Tung (Temple of Doi Tung Pagoda), Mae Fah Luang District, Chiang Rai, Thailand. Healthy roots, stems and leaves of *D.catenatum* were collected from Guizhou Province in China. Materials were packed in zip-lock bags or tubes containing silica gel on ice. Fungal isolation was carried out within 48 hours following collection.

### Fungal isolation and cultivation

Surface sterilization and endophyte isolation were carried out as described by Nontachaiyapoom et al. (2010) with some modifications. First, materials were washed with running water. Roots, stems and leaves were immersed in a solution containing 3% (v/v) H_2_O_2_ and 70% (v/v) ethanol for 5 minutes, and then rinsed with sterile distilled water for three times. Sterilized materials were cut into 2 mm^2^ and placed on potato dextrose agar (PDA) containing 50 μg/ml oxytetracycline, 50 μg/ml penicillin and 50 μg/ml streptomycin (Otero et al. 2002). Samples were incubated at 28 °C under natural light. Single spores were transferred to fresh PDA to obtain pure cultures. The pure cultures were deposited at China General Microbiological Culture Collection Center (CGMCC), International Collection of Micro-organisms from Plants (ICMP) and Mae Fah Luang University Culture Collection (MFLUCC). The dry cultures of new species were deposited in Mae Fah Luang University herbarium (MFLC).

### DNA extraction and amplification

DNA samples were prepared from mycelium of pure fungal culture using EZgene Fungal gDNA Kit (GD2416, Biomiga, USA) as described by the manufacturer. Amplification reactions were performed using reagents purchased from BIOMIGA (San Diego, USA). Each 20-μl amplification reaction contained 10 μl of 2*Bench Top Taq Master Mix (0.05 units/μl Taq DNA polymerase, 0.4 mM dNTPs and 4mM MgCl_2_); 2 μl forward and reverse primers; 1μl of DNA template and 7 μl of water. The primers used in this study were ITS1/ITS4 (White et al. 1990), GDF/GDR (Templeton et al. 1992), 512F/783R (Carbone and Kohn 1999) and BT2A/BT2B (Glass and Donaldson 1995; Maharachchikumbura et al. 2012). The thermal cycling programs are presented in Table 1. PCR products were sent to Invitrogen (USA), Sangon Biotech and Sino GenoMax (China) for purification and sequencing.

**Table 1.** PCR thermal cycling process.

		**PCR amplification**				
**Region/gene**	**Initial denaturation**	**Cycle number**	**Denaturation**	**Annealing**	**Elongation**	**Final elongation**
ITS	95 °C 3 min	30	95 °C 1 min	53 °C 1 min	72 °C 1 min	72 °C 10 min
GAPDH	95 °C 3 min	35	95 °C 1 min	60 °C 30 s	72 °C 45 s	
ACT	95 °C 3 min	40	94 °C 45 s	52 °C 30 s	72 °C 90 s	
ß-tubulin	95 °C 3 min	35	94 °C 1 min	55 °C 55 s	72 °C 1 min	

### Sequence analysis

Either single-directional sequencing results (for ITS and GAPDH) or bi -directional sequencing results (for ACT and TUB2) were manually trimmed and/or assembled into contigs using CodonCode aligner software (CodonCode Corporation, Dedham, Massachusetts). Through the latest publications and the observation for ML tree topology, a selected set of ITS, GAPDH, ACT and TUB2 sequences especially those of ex-type/ex-epitype materials used in the phylogenetic analysis were downloaded from GenBank (Table 2). Five datasets of *Colletotrichum* spp. ITS (134nt), GAPDH (113nt), ACT (119nt), ß–tubulin (125nt) and a concatenated dataset were constructed. Sequences were aligned using MAFFT version 6 (Katoh and Toh 2008; mafft. cbrc. jp/ alignment/server/). Aligned datasets were visually inspected and misaligned regions were manually edited where necessary using Bio-Edit version 7.2.5 (Hall 1999). Ambiguous regions were trimmed using trimAL version 1.3 (Capella–Gutierrez, Silla–Martinez and Gabaldon 2009) available online through Phylemon 2.0 (http://phylemon.bioinfo.cipf.es/). After trimming, the final alignments contained 578 sites for ITS, 298 sites for GAPDH, 290 sites for ACT and 480 sites for ß–tubulin. The concatenated dataset contained a total of 134 taxa and 1646 sites that were used for all subsequent analyses and submitted to TreeBase (http://purl.org/phylo/treebase/phylows/study/TB2:S22431). Gaps were treated as missing data in maximum likelihood (ML), Bayesian inference (BI) and parsimony trees. Parsimony trees were constructed with PAUP (Phylogenetic Analysis Using Parsimony) version 4.0 beta 10 (Swofford 2002). Heuristic searches were conducted as follows: 1000 starting trees were generated using stepwise addition and random addition sequence replicates, followed by branch swapping using the tree–bisection–reconnection (TBR) algorithm. The inferences for MP tree were length = 6732 steps, CI = 0.294, RI = 0.760, RC = 0.223, HI = 0.706. Maximum likelihood analyse was conducted with RAxMLGui 1.31 (Silvestro and Michalak 2012). The general time reversible (GTR) model of nucleotide substitution was used and the inverse gamma distribution option was implemented. The topology of the resulting tree was similar to that of the maximum parsimony tree. Bootstrap support was calculated from 1000 replicates, which were subsequently mapped onto the best-scoring ML tree. Bayesian inference trees were computed using MrBayes version 3.1.2 (Ronquist and Huelsenbeck 2003). The concatenated dataset was partitioned and the ultrafast bootstrap (Minh et al. 2013) implemented in the IQ-TREE software (Nguyen et al. 2015) as well as Mrmodeltest 2.3 (Nylander 2004) were used to estimate the best fitting models according to the Bayesian information criterion (BIC). The GTR model with inverse gamma distribution and HKY model with gamma distribution were used as the most appropriate for the ITS and GAPDH respectively. The Hasegawa, Kishino & Yano (HKY) model with inverse gamma distribution and GTR model with gamma distribution were selected for the ACT and ß-tubulin datasets. Two sets of four simultaneous independent chains of Markov chains Monte Carlo (MCMC) simulations were run for 6,000,000 generations, 25% of trees were discarded as burn-in and the remaining trees were used to calculate the posterior probabilities. Convergence was assumed when the standard deviation of split sequences was less than 0.01. The fungal isolates and sequences of region/genes used in *Colletotrichum* phylogenetic analysis are listed in Appendix A.

**Table 2.** *Colletotrichum* strains and species isolated from *Dendrobium* orchids.

Orchid sample	Sample site	Tissue	Number of fungal strains	*Colletotrichum* species	Code
* D. cariniferum *	Chiang Rai, Thailand	Root	0	0	–
		Stem	1	*** C. cariniferi ***	**MFLUCC 14-0100**
		Leaf	0	0	–
* D. harveyanum *	Chiang Rai, Thailand	Root	0	0	–
		Stem	0	0	–
		Leaf	2	* C. orchidophilum *	MFLUCC 14-0161 MFLUCC 14-0162
*Dendrobium* sp. 1	Chiang Rai, Thailand	Root	2	*** C. parallelophorum ***	**MFLUCC 14-0077 MFLUCC 14-0079**
		Stem	3	*** C. parallelophorum ***	**MFLUCC 14-0082 MFLUCC 14-0083 MFLUCC 14-0085**
		Leaf	4	*C.boninense*, *C.jiangxiense*, *C.fructicola*	MFLUCC 14-0086 MFLUCC 14-0087 MFLUCC 14-0091 MFLUCC 14-0092
*Dendrobium* sp. 2	Chiang Rai, Thailand	Root	2	***C.chiangraiense***; *C.fructicola*	**MFLUCC 14-0119**MFLUCC 15-0262
		Stem	3	*C.boninense*, ***C.watphraense***, *C.* sp. indet.	MFLUCC 15-0120 **MFLUCC 15-0123**MFLUCC 15-0124
		Leaf	3	*C.citricola*, ***C.doitungense***	**MFLUCC 15-0128**MFLUCC 15-0129 MFLUCC 15-0131
*Dendrobium* sp. 3	Chiang Rai, Thailand	Root	0	0	–
		Stem	0	0	–
		Leaf	1	* C. boninense *	MFLUCC 15-0148
* D. catenatum *	Xing Yi, China	Root	0	0	–
		Stem	0	0	–
		Leaf	1	* C. boninense *	MFLUCC 15-0261

“–” means that no strain was isolated. The strains of new species are in **bold** font.

### Morphological analysis

Sporulation of studied fungi was induced on thin pieces of Corn malt agar medium (CMA). The strains that did not sporulate on CMA were cultured on PDA or Sabouraud dextrose agar (SDA) with sterilized orchid tissues in order to induce sporulation. An autoclaved toothpick was placed on CMA for one strain *C.cariniferi* to induce sporulation. Cultures were grown in a dark cabinet at room temperature (28 °C) and observed for every seven days or less. The growth rate was evaluated when mycelia nearly covered the whole medium surface. Once an acervuli or ascomata were observed, photos were taken with a stereomicroscope (SteREO Discovery. V8, Carl Zeiss Microscopy GmBH, Germany). Cross-sections and conidiomata crushed in water were observed under a compound microscope (EOS 600D, Nikon, Japan). Ascomata and conidiomata were observed under a Motic SMZ–140 microscope (China). Conidiophore, conidia, appressoria, ascomata, asci, ascospores and other visible structures such as chlamydospore were used for evaluating morphological characteristics in this study (Damm et al. 2014). The recommendations of Jeewon and Hyde (2016) were followed in establishing new species.

## Results

### Fungal isolation and Identification

Twenty-two endophytic *Colletotrichum* strains were isolated from six *Dendrobium* species (Table 2). The highest number of *Colletotrichum* strains and species were isolated from *Dendrobium* sp. 1 followed by *Dendrobium* sp. 2. All three tissue types of the two orchids hosted at least one strains of *Colletotrichum*. Among the three tissue types, the highest number of *Colletotrichum* strains and species were isolated from leaves. *Colletotrichumboninense* and *C.fructicola* were respectively the most frequently isolated *Colletotrichum* species. Interestingly, *C.boninense* was isolated from *Dendrobium* species collected from both geographical areas studied (i.e., Chiang Rai, Thailand and Guizhou, China).

**Figure 1.**
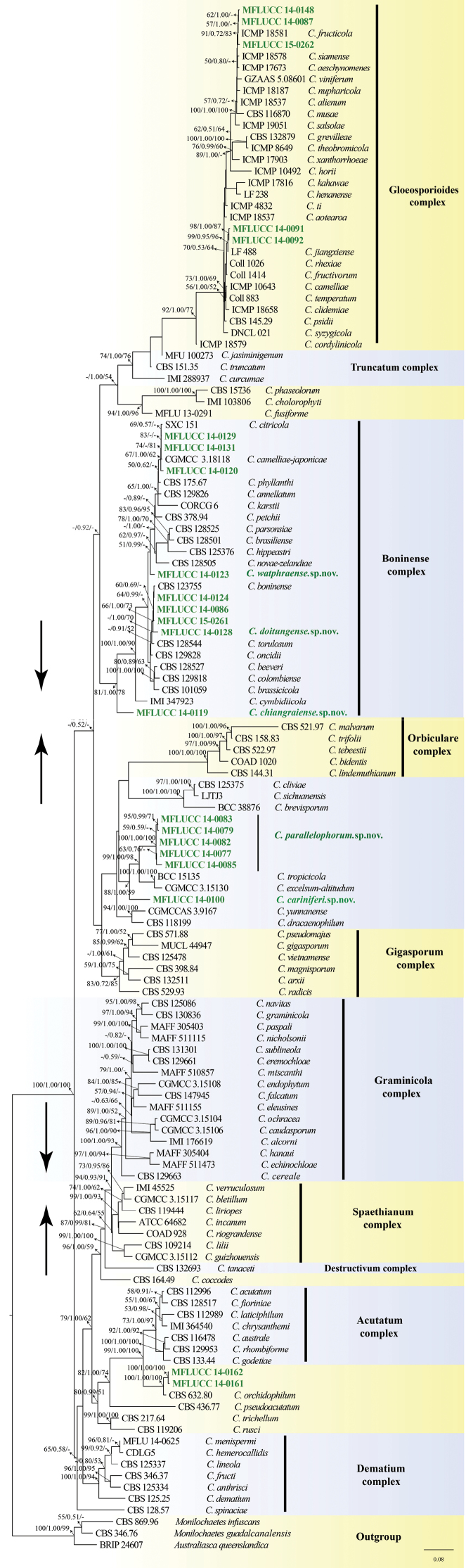
Maximum likelihood (ML) tree of *Colletotrichum* inferred from 134 taxa and 1646 sites from a concatenated dataset containing ITS, GAPDH, ACT and ß-tubulin sequence data. Values at nodes indicate bootstrap percentages (BP) for ML, Bayesian posterior probabilities (PP) and BP for maximum parsimony (MP) in this order. Only BP over 50%, PP over 0.50 and MP over 50 are shown. Dashes correspond to lower than the above-mentioned values. The isolated fungal endophytes in this study are shown in green **bold** text. Scale bar corresponds to 0.08 substitutions per site. “*” indicates the new species.

### Sporulation results

All *Colletotrichum* strains could grow on three kinds of media. *Colletotrichumcitricola, C.doitungense*, *C.fructicola* and *C.parallelophorum* produced both sexual and asexual morphs in culture. *Colletotrichumboninense*, *C.cariniferi*, *C.orchidophilum* and *C.watphraense* produced only the asexual morph and *C.chiangraiense* produced only sexual morph in culture. Measurements of important vegetative and reproductive characteristics of isolated strains are given in Table 3.

**Table 3.** Synopsis of size (µm) of structures of Colletotrichum species identified in this study.

				Sexual morph			Asexual morph		
*Colletotrichum* species	N	Vegetative hyphae diam (µm)	Setae (µm)	Ascomata (µm)	Size of asci (µm)	Size of ascospores (µm)	Size of conidiomata (µm)	Size of conidiophore (µm)	Size of conidia (µm)
*C.cariniferi* sp. nov.	1	3.5–8.2	–	–	–	–	50 ×50	(37.5–) 42.3–65 (–71.6) × (3.1–) 3.8–5.9 (–6)	(24.1–) 26.8–33.0 (–36.1) × (7.9–) 8.3–9.6 (–10.2), L/W=3.4
*C.chiangraiense* sp. nov.	1	4.6.± 1.8	–	(14.4–) 15.3–19.6 (–20.5) × (7.4–) 7.3–7.9 (–8)	(30.7–) 33.4–52.7 (–72.2) × (5.7–) 6.5–8.2 (–9.4)	(11–) 11.9–15.4 (–16.7) × (2.2–) 2.8–3.8 (–4.4), L/W=4.2	–	–	–
* C. citricola *	3	3.1 ± 1.1	(51.8–) 54.1–67.8 (–68.5) × (2.3–) 2.4–5.8 (–7.2)	(34.5–) 46.4–84.9 (–87.1) × (31.7–) 33.8–46.5 (–50.9)	(41.3–) 49.4–65.0 (–71.6) × (8.3–) 9.5–12.9 (–14.3)	(14.4–) 14.8–17.5 (–19.3) × (5.4–) 5.7–7.1 (–7.6), L/W = 2.5	–	(10.8–) 16.7–25.6 (–30.6) × (3.1–) 4–5.3 (–5.6)	(12.5–) 13.4–15 (16.5–) × (5–) 5.9–6.9 (–7.2), L/W = 2.2
*C.doitungense* sp.nov.	2	1.1–3.5	–	(125.5–) 126.9–133.7 (–135.1) × (101.3–) 101.8–104.3 (–104.8)	(51.1–) 53.7–70.6 (–71.6) × (8.5–) 8.8–10.1 (–10.4)	(16.1–) 17.5–21.5 (–23.4) × (4.5–) 5.1–7(–7.5), L/W=3.2	–	(9.1–) 14.3–26.8 (–29.4) × (3–) 3.1–4.5 (–5)	(6.6–) 8.6–13.8 (–15) × (2.6–) 3.8–8.9 (–13.8), L/W=1.75
* C. fructicola *	3	2.6–5	(53–) 57.2–73.1 (–83.3) × (3.4–) 3.5–4 (–4.1)	(131.9–) 138.4–163.6 (–171.5) × (120.9–) 123.6–142.1 (–143.2)	(57.6–) 61.2–82.6 (–94.3) × (8.7–) 9.3–13.3 (–15.8)	(10–) 12.0–20.0 (–20.9) × (3.6–) 4.1–5.2 (–5.3), L/W=3.4	500×400	–	(12.8–) 13.8–16.6 (–18.6) × (2.7–) 3.5–7.8 (–16), L/W=2.9
* C. jiangxiense *	2	1.3–2.1	–	–	–	–	–	(12.7–) 13.5–21.4 (–23.4) × (1.9–) 2–3 (–3.2)	(8.6–) 9–12.4 (–13.2) × (3.5–) 3.6–4.4 (–4.5), L/W=2.6
* C. orchidophilum *	2	1.9–5.4	–	–	–	–	200×300	–	(9.6–) 11.7–14.1 (–14.7) × (2.9–) 3.5–4.4 (–4.8), L/W=3.3
*C.parallelophorum* sp.nov.	2	2–4.3	(56.7–) 60.2–79.2 (–81.2) × (2.8–) 2.9–3.7 (–3.9)	(267–) 261.4–342.3 (–346.2) × (190.4–) 173–272.5 (–280)	(43.3–) 44.1–63.3 (–66.5) × (7.6–) 8–9.8 (–10)	(13.9–) 14.1–18 (–20.9) × (3.1–) 3.9–5.4 (–5.8), L/W=3.5	200×200	(18.3–) 20.82–34 (–41.2) × (2.6–) 2.8–4.3 (–5.4)	(12.1–) 13.8–16.8 (–18.9) × (3.3–) 4.4–7.5 (–7.9), L/W=2.6
*C.watphraense* sp. nov.	1	1.6–4.3	–	–	–	–	200×300	(15.8–) 18.5–26.8 (–29.1) × (3.4–) 3.8–5.1 (–5.7)	(12.4–) 12.5–14.6 (–15.2) × (4.4–) 4.5–5.8 (–6.1), L/W=2.3

*”N”means the number of cultures that were used in measuring the characteristics.

## Phylogenetic results

### Phylogenetic analyses

All the sequences of ITS, GAPDH, ACT and β-tubulin of 22 strains of *Colletotrichum* obtained in this study were deposited in GenBank (List in Appendix B). The three selected outgroup species (i.e. *Australiascaqueenslandica* BRIP 24607; *Monilochaetesinfuscans* CBS 869.96 and *Monilochaetesguadalcanalensis* CBS 346.76) formed a strongly supported cluster (100ML/1.00BI/99MP). The ingroup consisted of all *Colletotrichum* sequences and was fully supported by all three methods of analysis (100ML/1.00BI/100MP). Five strains grouped within the gloeosporioides complex: MFLUCC 14-0087, MFLCCC 14-0091, MFLUCC 14-0092,

MFLUCC 14-0148 and MFLUCC 14-0262. The sequences of MFLCCC 14-0091 and MFLUCC 14-0092 were nearly identical and close to *C.jiangxiense* with strong support (99ML/0.95BI/96MP). MFLUCC 14-0087, MFLUCC 14-0148 and MFLUCC 15-0262 clustered with *C.fructicola* (ICMP 181873) (91ML/0.72BI/83MP).

Nine of the newly sequenced strains clustered within the boninense species complex: MFLUC 14-0086, MFLUCC 14-0119, MFLUCC 14-0120, MFLUCC 14-0123, MFLUCC 14-0124, MFLUCC 14-0128, MFLUCC 14-0129, MFLUCC 14-0131, MFLUCC 15-0261. MFLUCC 14-0086, MFLUCC 14-0124 and MFLUCC 14-0261 shared very similar sequences. MFLUCC 14-0128 grouped as sister to the three above-mentioned strains (66ML/1.00BI/73MP). MFLUCC 14-0123 formed a separated clade from other species by only Bayesian analysis (1.00BI). MFLUCC 14-0120, MFLUCC 14-0129 and MFLUCC 14-0131 formed a cluster with *C.camelliae-japonicae* and *C.fructicola* (76ML/1.00BI/62MP). MFLUCC 14-0120 and MFLUCC 14-0129 differed by only three base pairs in trimmed concatenated alignment. MFULCC 14-0119 was placed basally to the boninense species complex with strong support (100ML/0.96BI/90MP).

MFLUCC 14-0161 and MFLUCC 14-0162 grouped outside the currently accepted species complexes. The two had a close relationship and formed a clade with *C.orchidophilum*, which is a singleton and a sister taxon to the acutatum species complex. They hold the maximum support with all three methods of analysis. *Colletotrichumorchidophilum* differed 1.5% and 1.3% with MFLUCC 14-0161 and MFLUCC 14-0162 respectively. MFLUCC 14-0077, MFLUCC 14-0079, MFLUCC 14-0082, MFLUCC14–0083 and MFLUCC 14-0085 formed a novel clade (100ML/1.00BI/100MP), which grouped as sister clade to the *C.excelsum-altitudum*/*C.tropicicola* clade and MFLUCC 14-0100 (88ML/1.00BI/59MP). MFLUCC 14-0100 took a solo branch in the basal position among them (99ML/1.0BI/98MP).

## Taxonomy

The 22 strains isolated as endophytes were assigned to eleven species, five known species, five new species and one undetermined species. We obtained the sexual and asexual morphs for four strains. The sexual morph only was obtained in the case of *C.chiangraiense.* The descriptions of the fungal endophytes identified in this study are as follows.

### Colletotrichum cariniferi

Taxon classificationFungiGlomerellalesGlomerellaceae

X.Y. Ma, K.D. Hyde & Jayawardena sp.nov.

#### Etymology.

In reference to the host epithet cariniferum.

#### Holotype.

MFLC 17-1199 (ex-holotype culture: MFLUCC 14-0100).

#### Description.

*Sexual morph* not observed.

Asexual morph on CMA. Vegetative hyphae 3.5–8.2 µm diam (N=20), hyaline to brown, smooth-walled, septate, branched. Appressoria (9.7–) 10.4–17 (–20.5) × (6.5–) 7.1–11.3 (–13.6) µm (N=6), globose to sub-globose, light brown. Conidiomata 50 × 50 µm (N=10), developing with mycelia, globose to irregular, milk orange to orange, in mass brown. Conidiophores (37.5–) 42.3–65 (–71.6) × (3.1–) 3.8–5.9 (–6) µm (N=6), smooth-walled, unbranched, hyaline. The part connected with conidia of conidiogenous cell inflated and some with large guttules. Conidia (24.1–) 26.8–33.0 (–36.1) × (7.9–) 8.3–9.6 (–10.2) µm (N=30), L/W = 3.4, ellipsoidal to cylindrical, with one end inflated when immature state, both ends rounded when mature, with 2 to 3 guttules, hyaline.

Cultures on CMA flat with entire margin. Growth rate: 0.23cm/day, with 50-days for sporulation. Cottony, pale cinnamon to light brown, scattered pale mycelia in spots around the middle inoculum clump, sometimes covered short, floccose-felty, white, aerial mycelium, reverse buff brown.

**Figure 2.**
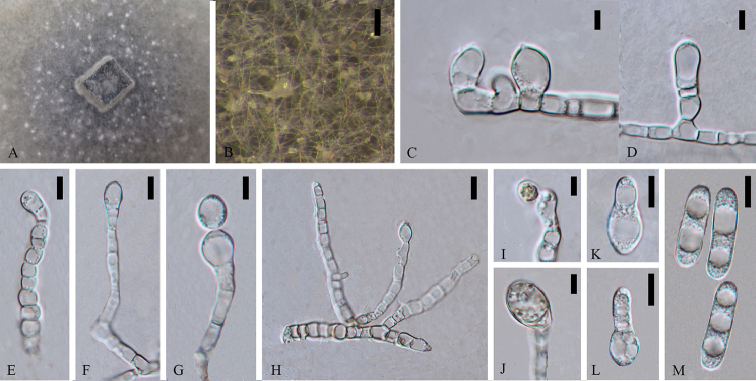
*Colletotrichumcariniferi* (holotype). **A** Colony **B** Conidiomata **C, I–J** Appressoria **D–H** Conidiophores **K–M** Conidia. Scale bars: 100 µm (**B**), 5 µm (**C–D**), 10 µm (**E–H**), 5 µm (**I–M**).

#### Material examined.

Thailand, Chiang Rai, Wat Phra That Doi Tung (Temple of Doi Tung Pagodas), the host *Dendrobiumcariniferum* was sampled on 19 December 2013, Collector: Sureeporn Nontachaiyapoom, Natdanai Aewsakul, Xiaoya Ma.

#### Notes.

*Colletotrichumcariniferi* (MFLUCC 14-0100) clusters in a separate branch with a good support (88ML/1.00BI/59MP). The species is most phylogenetically close to *Colletotrichumexcelsum-altitudum* and *C.tropicicola*, but they are morphologically different. *C.cariniferi* holds 77 and 91 different base pairs compared with *C.tropicicola* and *C.excelsum-altitudum* respectively. *Colletotrichumcariniferi* has much larger conidia than that of closely related strains in the tree (conidia (24.1–) 26.8–33 (–36.1) × (7.9–) 8.3–9.6 (–10.2) µm (N=30), L/W = 3.4 vs. conidia of *C.tropicicola* 13–16.5×5–7 μm and *C.excelsum-altitudum* 14.8 ± 0.8 × 5.8 ± 0.4 μm) (Noireung 2012; Tao et al. 2013). Blastn searches with sequence of MFLUCC 14-0100 resulted in 100% matches with ITS sequence of endophytic *Colletotrichum* sp. strain S4 isolated from *Dendrobuimnobile* in China (GenBank FJ042517, Yuan et al. 2009) and 96% identity with TUB2 sequences of *C.arxii* strain CBS 169.59 isolated from *Oncidiumexcavatum* (GenBank KF687868, Liu et al. 2014) in Netherlands and another *C.arxii* strain CBS 132511 isolated from *Paphlopedilum* sp. in Germany (GenBank KF687881, Liu et al. 2014) respectively. *Colletotrichumcariniferi* from stems of *D.cariniferum* is introduced as a new species.

### Colletotrichum chiangraiense

Taxon classificationFungiGlomerellalesGlomerellaceae

X.Y. Ma, K.D. Hyde & Jayawardena sp.nov.

Figure 3

#### Etymology.

In reference to the host sample site Chiang Rai, Thailand.

#### Holotype.

MFLU 17-1201 (ex-holotype culture: MFLUCC 14-0119).

#### Description.

*Asexual morph* not observed.

Sexual morph on CMA. Vegetative hyphae 4.6.± 1.8 µm diam (N=20), hyaline to pale brown, smooth-walled, septate, branched. Chlamydospore globose, brown. Hyphae fusion and crozier observed. Ascomata rare, covered by mycelia, black. Appressoria (14.4–) 15.3–19.6 (–20.5) × (7.4–) 7.3–7.9 (–8) µm (N=2), single, outline ampulliform or ovate, pale brown. Asci (30.7–) 33.4–52.7 (–72.18) × (5.7–) 6.5–8.2 (–9.4) µm (N=15), cylindrical, straight to curved, unitunicate, 8–spored. Ascospores (11–) 11.9–15.4 (–16.7) × (2.2–) 2.8–3.8 (–4.4) µm (N=20), L/W = 4.2, bi-seriately, smooth-walled, cylindrical or fusiform, one guttule in the middle, hyaline.

Cultures on CMA flat with entire margin. Growth rate: 0.6cm/day, with 20-days for sporulation. Fluffy, dark green in the middle and white margin, reverse black in the middle.

**Figure 3.**
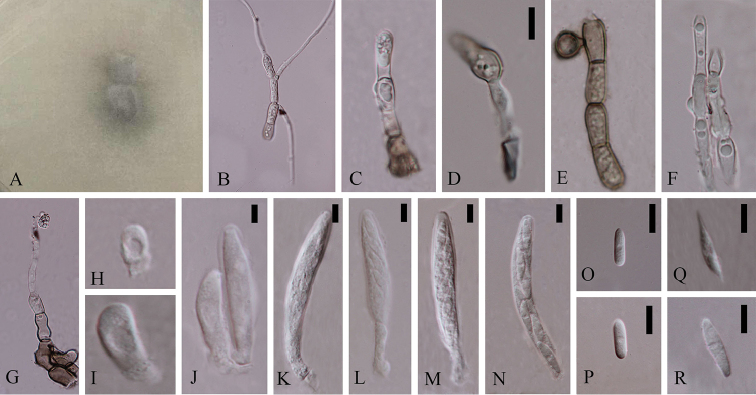
*Colletotrichumchiangraiense* (holotype). **A** Colony **B** Spore germination **C** Conidiophore **D** Appressoria **E** Chlamydospore **F** Mycelia fusion **G** Crozier **H–N** Asci **O–R** Ascospores. Scale bars: 20 µm (**D**), 20 µm (**G**), 5 µm (**J–N**), 10 µm (**O–R**).

#### Material examined.

Thailand, Chiang Rai, Wat Phra That Doi Tung (Temple of Doi Tung Pagodas), the host *Dendrobium* sp. 2 was collected on 19 December 2013, Collector: Sureeporn Nontachaiyapoom, Natdanai Aewsakul, Xiaoya Ma.

#### Notes.

*Colletotrichumchiangraiense* (MFLUCC 14-0119) formed a single branch with the support of 81ML/1.00BI/78MP in boninense species complex. It has 125 different base pairs (mainly in ITS and ACT) from the closest strain *C.cymbidiicola*. Blastn searches with sequences of MFLUCC 14-0119 resulted in 99% identity with ITS of the endophytic *C.crassipes* strain DO93 (GenBank KP050648) isolated from *Dendrobiumofficinale* in China (Unpublished), 99% identity with ACT of the endophytic *Colletotrichum* sp. strain COAD 2105 (GenBank KY407893) isolated from *Cattleyajongheana* in Brazil (Unpublished), 98% identity with TUB2 of the endophytic *C.boninense* strain CBS 125502 (GenBank KJ955336) isolated from *Camelliasinensis* in China (Liu et al. 2015) and 98% identity with TUB2 of the endophytic *C.boninense* strain CGMCC 3.15165 (GenBank KC244156) isolated from *Bletillaochracea* in China (Tao et al. 2013). This species was observed antheridium, mycelia fusion and crozier, which indicates that this species may be homothallic. Here we introduce the strain isolated from root of *Dendrobium* sp. 2 as a new species.

### Colletotrichum watphraense

Taxon classificationFungiGlomerellalesGlomerellaceae

X.Y. Ma, K.D. Hyde & Jayawardena sp. nov.

Figure 4

#### Etymology.

In reference to the host sample site – Wat Phra temple in Chiang Rai, Thailand.

#### Holotype.

MFLU 17-1202 (ex-holotype culture: MFLUCC 14-0123).

#### Description.

*Sexual morph* not observed.

Asexual morph on CMA. Vegetative hyphae 1.6–4.3 µm diam (N=20), smooth-walled, septate, branched, hyaline. Chlamydospores and appressoria not observed. Conidiomata 200 × 300 µm, brown, Conidiophores (15.8–) 18.5–26.8 (–29.1) × (3.4–) 3.8–5.1 (–5.7) µm (N=16), smooth-walled, septate, branched or single, periclinal thickening, hyaline. Conidia (12.4–) 12.5–14.6 (–15.2) × (4.4–) 4.5–5.8 (–6.1) µm (N=5), L/W = 2.3, aseptate, ellipsoidal, single guttules in the middle, the one part inflated, hyaline.

Cultures on CMA flat with entire margin. Growth rate: 0.45cm/day, with 30-days for sporulation. Fluffy, white to light buff orange. Perithecia isolated. Acervuli under white cotton-like mycelia, irregular, asymmetrical surface, light brown to brown.

**Figure 4.**
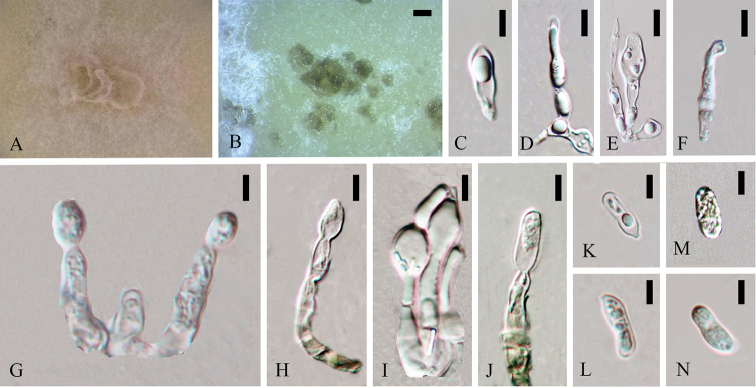
*Colletotrichumwatphraense* (holotype). **A** Colony **B** Fruiting body **C–J** Conidiophores **K–N** Conidia. Scale bars: 200 µm (**B**), 5 µm (**C–N**).

#### Material examined.

Thailand, Chiang Rai, Wat Phra That Doi Tung (Temple of Doi Tung Pagodas), the host *Dendrobium* sp. 2 was collected on 19 December 2013, Collector: Sureeporn Nontachaiyapoom, Natdanai Aewsakul, Xiaoya Ma.

#### Note.

MFLUCC 14-0123 formed a singular branch with other species and only supported by 1.00BI in boninense species complex. There were 42bp (2.6%) and 85bp (5.2%) differences in GAPDH between *Colletotrichumwatphraense* and its close strains *Colletotrichumboninense*/*C.novae-zelandiae* respectively. The closest matches in a blastn search with ITS sequences of the strain MFLUCC 14-0123 are *C.cymbidiicola* strain FS21 (GenBank KP689224) iaolated from a rare medical plant *Huperziaserrata* with 99% identity in China (Wang et al. 2016), *C.gloeosporioides* strain Trtsf02 (GenBank GU479899) isolated from *Trillumtschonoskii* with 99% identity in China (Unpublished) and pathogenic *C.boninense* strain CO5016 (GenBank GU935883) isolated from ginseng with 99% identity in Korea (Unpublished). GAPDH and ACT sequences blastn results showed its closest matches are pathogenic *C.citricola* strain SXC 151 (GenBank KC293736) isolated from Proteaceae with 99% identity in Netherlands (Liu et al. 2012) and *C.boninense* strain CBS 125502 (GenBank KJ954462) isolated from *Camellia* sp. with 99% identity in unknown locality (Liu et al. 2015). Blastn search with TUB2 sequence results in 99% identity with two *C.boninense* strains CBS 125502 (GenBank KJ955336) and the strain CGMCC 3.15165 (GenBank KC244156) as mentioned above. The conidiophores were much longer (40 µm long) in *C.boninense*. Conidia of the strain CBS 123755 have straight, cylindrical to clavate, conidia with a rounded apex; and base with a prominent hilum, sometimes with two large polar guttules, which is different from *Colletotrichumwatphraense*. Here we assigned the strain isolated from stem of *Dendrobium* sp. 2 as a new species.

### Colletotrichum doitungense

Taxon classificationFungiGlomerellalesGlomerellaceae

X.Y. Ma, K.D. Hyde & Jayawardena sp.nov.

Figure 5

#### Etymology.

In reference to the host sample site Doi tung, Chiang Rai, Thailand.

#### Holotype.

MFLU 17-1200 (ex-holotype culture: MFLUCC 14-0128).

#### Description.

Asexual morph on CMA. Vegetative hyphae 1.1–3.5 µm diam, hyaline, smooth-walled, septate, branched. Setae and chlamydospores not observed. Conidiomata and ascomata cluster together. Conidiophores (9.1–) 14.3–26.8 (–29.4) × (3–) 3.1–4.5 (–5) µm, smooth-walled, unbranched, septate, constricted septum, hyaline. Conidiogenous cell (3.1–) 3.2–5.8 (–7.5) × (2.6–) 3–4 (–4.5) µm (N=14), globose to sub-globose, smooth-walled, hyaline. Conidia (6.6–) 8.6–13.8 (–15) × (2.6–) 3.8–8.9 (–13.8) µm (N=22), L/W = 1.75, globose to ellipsoidal, both ends rounded, smooth-walled, hyaline.

Sexual morph on CMA. Ascomata (125.5–) 126.9–133.7 (–135.1) × (101.3–) 101.8–104.3 (–104.8) µm (N=10), sub-globose, closed, pale brown to brown. Peridium 3–11.5 µm thick, Asci (51.1–) 53.7–70.6 (–71.6) × (8.5–) 8.8–10.1 (–10.4) µm (N=8), cylindrical, slight curved, composed of pale to medium brown flattened angular cells, unitunicate, smooth-walled, 8–spored, hyaline. Ascospores (16.1–) 17.5–21.5 (–23.4) × (4.5–) 5.1–7.0 (–7.5) µm (N=20), L/W = 3.2, fusiform, blunt to somewhat acute or acute both ends, single guttule in the middle, septate, bi-seriately, smooth-walled, hyaline.

Cultures on CMA flat with entire margin. Fluffy, white, reverse same. Growth rate: 0.6cm/day, with 20-days for sporulation. Brown ring in the middle. Perithecia gregarious. Acervuli and ascomata in mass light brown to brown.

**Figure 5.**
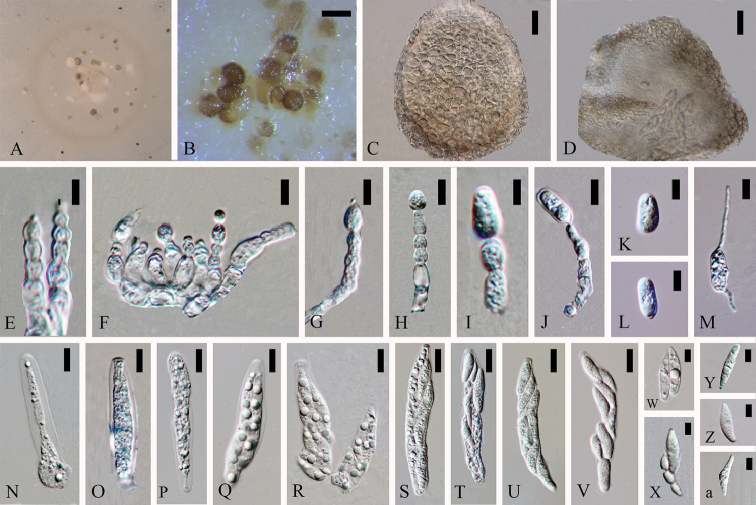
*Colletotrichumdoitungense* (holotype). **A** Colony **B** Fruiting body **C–D** Ascomata **E–J** Conidiophores **K–L** Conidia **M** Spore germination **N–V** Asci **W–a** Ascospores. Scale bars: 100 µm (**B**), 20 µm (**C–D**), 5 µm (**E–M**), 10 µm (**N–V**), 5 µm (**W–a**).

#### Material examined.

Thailand, Chiang Rai, Wat Phra That Doi Tung (Temple of Doi Tung Pagodas), the host *Dendrobium* sp. 2 was collected on 19 December 2013, Collector: Sureeporn Nontachaiyapoom, Natdanai Aewsakul, Xiaoya Ma.

#### Notes.

*Colletotrichumdoitungense* form an independent lineage from other strains with good support (66ML/1.00BI/73MP) in boninense species complex. The ITS sequence of MFLUCC 14-0128 100% matches with unpublished pathogenic *C.cymbidiicola* strain OORC18 (GenBank JX902424) isolated from orchid in India and *C.karstii* strain R001 (GenBank JN715846) isolated from blackberry in Colombia (Unpublished). Blastn researches with sequences of MFLUCC 14-0128 results in 98% identity with GAPDH sequence of endophytic *C.boninense* strain CGMCC 3.15168 (GenBank KC843491) isolated from *Bletillaochracea* in China (Tao et al. 2013), 99% identity with ACT sequence of *C.boninense* strain CBS 125502 (GenBank KJ954462) and 99% identity with TUB2 sequence of *C.citricola* strain SXC 151 (GenBank KC293656) as mentioned above. Its conidiogenus cell is globose to sub-globose, which differ from cylindrical to ellipsoidal conidiogenus cell in *C.boninense* (Damm et al. 2012). This strain has 2 and 0 in ITS, 6 and 1 in GAPDH, 3 and 2 in ACT, 17 and 16 base pair differences from its sister taxon *C.torulosum* and MFLUCC 14-0261 respectively. Here we introduce *Colletotrichumdoitungense* isolated from root of *Dendrobium* sp. 2 as a new species.

### Colletotrichum parallelophorum

Taxon classificationFungiGlomerellalesGlomerellaceae

X.Y. Ma, K.D. Hyde & Jayawardena sp. nov.

Figure 6

#### Etymology.

In reference to the parallel conidiophores.

#### Holotype.

MFLU 17-1198 (ex-holotype culture: MFLUCC 14-0083).

#### Description.

Asexual morph on CMA. Vegetative hyphae 2–4.3 µm diam (N=30), smooth-walled, septate, branched, hyaline to pale brown. Chlamydospores not observed. Conidiomata acervular, orange. Appressoria (56.7–) 60.2–79.2 (–81.2) × (2.8–) 2.9–3.7 (–3.9) µm (N=8), single, sub-globose, brown, rare. Conidiophores and setae formed on a cushion of pale brown cells (1.9–) 2.4–4 (–4.6) µm diam. Setae medium brown, smooth-walled, 2 or 3-septate; base cylindrical, constricted at the base, apex acute. Conidiophores (18.3–) 20.8–34 (–41.2) × (2.6–) 2.8–4.3 (–5.4) µm (N=20), smooth-walled, 2 to 3-septate, branched, hyaline to pale brown. Conidiophores and setae formed on a cushion of pale brown prismatic cells, sometimes with guttules. Conidia (12.1–) 13.8–16.8 (–18.9) × (3.3–) 4.4–7.5 (–7.9) µm (N=50), L/W = 2.6, hyaline, smooth-walled, with 1 to 4 guttules, cylindrical with both ends rounded.

Sexual morph on CMA. Ascomata (267–) 261.4–342.3 (–346.2) × (190.4–) 173.0–272.5 (–280) µm (N=3), globose, glabrous, Ascomata isolated, scattered, irregular and asymmetrical, black. Peridium 13.6–46.4 µm thick, consist of pale to medium brown flattened angular cells. Ascogenous hyphae hyaline, smooth-walled. Asci (43.3–) 44.1–63.3 (–66.5) × (7.6–) 8.0–9.8 (–10) µm (N=7), cylindrical, straight, unitunicate, 8–spored. Ascospores (13.9–) 14.1–18 (–20.9) × (3.1–) 3.9–5.4 (–5.8) µm (N=23), L/W = 3.5, uni-to bi-seriately, aseptate, smooth-walled, ellipsoidal, single guttules in the middle, both ends rounded, hyaline.

Cultures on CMA flat with entire margin. Growth rate: 0.4cm/d, with 20-days for sporulation. With fluffy, light green and white mycelia. Ascomata sometimes growing together with acervuli.

**Figure 6.**
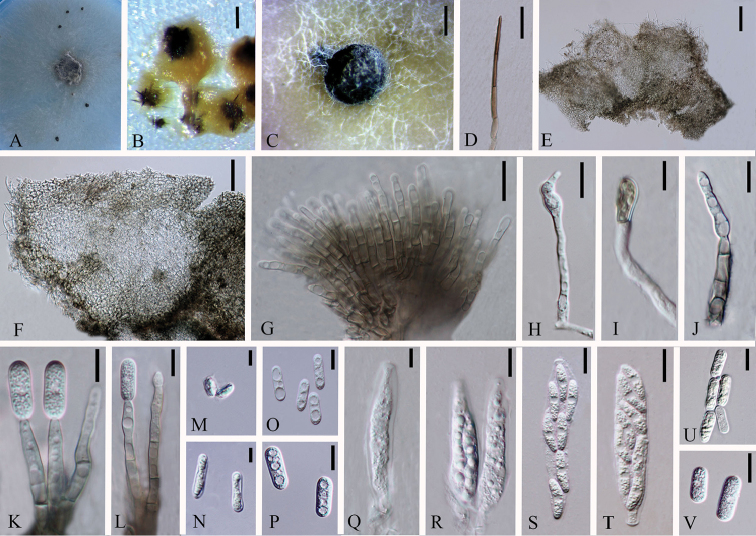
*Colletotrichumparallelophorum* (holotype). **A** Colony **B, C** Fruiting body **D** Setae **E–F** Ascomata **G, J–L** Conidiophores **I** Appressoria **M–P** Conidia **Q–T** Asci **U–V** Ascospore. Scale bars: 50 µm (**B**), 500 µm (**C**), 20 µm (**D**), 100 µm (**E**), 50 µm (**F**), 20 µm (**G**), 10 µm (**H–L**), 5 µm (**M, N**), 10 µm (**O–V**).

#### Material examined.

Thailand, Chiang Rai, Wat Phra That Doi Tung (Temple of Doi Tung Pagodas), the host *Dendrobium* sp. 1 was collected on 19 December 2013, Collector: Sureeporn Nontachaiyapoom, Natdanai Aewsakul, Xiaoya Ma.

#### Notes.

Strains MFLUCC 14-0077, MFLUCC 14-0079 and MFLUCC 14-0083 had identical sequence data and they formed a single clade with MFLUCC 14-0082 and MFLUCC 14-0085. They are closely related to *Colletotrichumexcelsum-altitudum* and *C.tropicicola*. MFLUCC 14-0077, MFLUCC 14-0079, MFLUCC 14-0082, MFLUCC 14-0083 and MFLUCC 14-0085 have similar morphological characteristics. Therefore, the five strains are regarded as the same species. There were totally 103bp and 101bp differences between MFLUCC 14-0083 and *C.excelsum-altitudum*/*C.tropicicola* respectively (mainly in GAPDH). Blastn researches with four-gene sequences of five strains presented 99% identity with ITS sequence of *C.cordylinicola* strain LC0886, 80% identity with GAPDH (GenBank JN050229), 90% identity with ACT (GenBank JN050218) and 93% identity with TUB2 (GenBank JN050246) sequences of *C.tropicola* strain LC0598 respectively as mentioned above. Conidia size and shape were very similar among MFLUCC 14-0083, *C.excelsum-altitudum* and *C.tropicicola*. Appressoria were rare and in strain MFLUCC–14–0083 appressoria were not variable like that in *C.excelsum-altitudum* and *C.tropicicola*. Here we introduced strains MFLUCC 14-0077, MFLUCC 14-0079, MFLUCC 14-0082 and MFLUCC 14-0083 and MFLUCC–14–0085 isolated from stems and roots of *Dendrobium* sp. 1 as *Colletotrichumparallelophorum* sp.nov.

### Colletotrichum citricola

Taxon classificationFungiGlomerellalesGlomerellaceae

F. Huang, L. Cai, K.D. Hyde & H.Y. Li

Figure 7

#### Description.

Asexual morph on CMA. Vegetative hyphae 3.1 ± 1.1 µm diam (N=20), smooth-walled, septate, branched, hyaline. Chlamydospores globose, hyaline. Conidiomata ovoid, orange. Setae (51.8–) 54.1–67.8 (–68.5) × (2.3–) 2.4–5.8 (–7.2) µm (N=6), smooth-walled, 1 or 3–septate, contracted to slightly inflated base, tapering to the apex, apex acute, pale brown to brown. Conidiophores (10.8–) 16.7–25.6 (–30.6) × (3.1–) 4–5.3 (–5.6) µm (N=27), smooth-walled, septate, hyaline. Conidia (12.5–) 13.4–15 (16.5–) × (5–) 5.9–6.9 (–7.2) µm (N=40), L/W = 2.2, ellipsoidal, smooth-walled, hyaline.

Sexual morph on CMA. Ascomata (34.5–) 46.4–84.9 (–87.1) × (31.7–) 33.8–46.5 (–50.9) µm (N=5), globose, ostiolate, clustered, pale brown to dark brown. Peridium 1.7–5.8 µm thick, composed of pale to medium brown, flattened, angular cells. Ascogenous hyphae hyaline, smooth-walled. Asci (41.3–) 49.4–65 (–71.6) × (8.3–) 9.5–12.9 (–14.3) µm (N=36), cylindrical, unitunicate, straight or curved, 8–spored. Ascospores (14.4–) 14.8–17.5 (–19.3) × (5.4–) 5.7–7.1 (–7.6) µm (N=25), L/W = 2.5, uni-or bi-seriately, smooth-walled, hyaline, fusiform or one end slightly rounded, with a single guttule in the middle.

Cultures on CMA flat with entire margin. Growth rate: 0.6cm/day, with18-days for sporulation. Fluffy, pale mycelia float on the dark scarlet pigment medium, reverse dark brown. Perithecia gregarious. Orange acervuli and ascomata in mass form thick globules.

**Figure 7.**
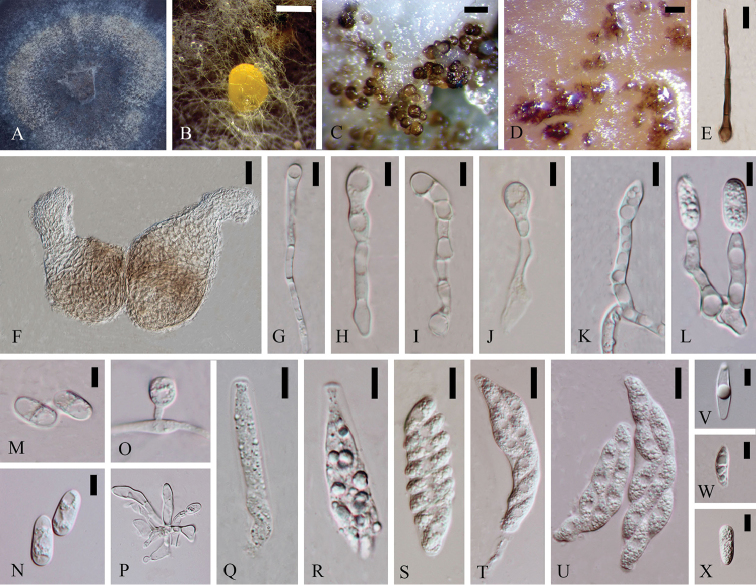
*Colletotrichumcitricola*. **A** Colony **B** Conidiomata **C** Fruiting bodies **D** Fruiting body with setae **E** Setae **F** Ascomata **G–L** Conidiophores **M, N** Conidia **O** Chlamydospore **P–U** Young asci **V–X** Ascospores. Scale bars: 500 µm (**B**), 200 µm (**C**), 10 µm (**E–F**), 5 µm (**G**), 5 µm (**M–N**), 10 µm (**Q–U**), 5 µm (**V–X**).

#### Notes.

Strains MFLUCC 14-0129 and MFLUCC 14-0131 had similar sequence data, cultures and conidia. There were 5bp and 7bp difference between the strains and *Colletotrichumcamelliae-japonicae* and *C.citricola* respectively. ITS sequence is 99% identity with unpublished *C.boninense* strain LD3–8–1 isolated from strawberry in China (Unpublished). Blastn searches sequences results in GAPDH (GenBank KC293736) and TUB2 (GenBank KC293656) sequences of *C.citricola* strain SXC 151 as mention above. ACT sequence is closest to *C.karstii* strain 42a (GenBank KT122921) isolated from *Coffeaarabica* in Mexico (Cristobal-Martinez et al. 2016). All morphological characteristics of the two strains were nearly the same as the protologue of *C.citricola.* Therefore, we name strains MFLUCC 14-0129 and MFLUCC 14-0131 as *C.citricola*. When compared with *C.camelliae-japonicae* (conidia: 11–14.5 ×5–6.5μm, mean ±SD = 12.5 ±0.8 ×5.5 ±0.3μm, L/W=1.5; ascospores: 13.5–18.5 ×4–5.5 μm, mean ± SD = 16.5 ±1.1×5±0.4μm, L/W = 3.3), strains MFLUCC 14-0129 and MFLUCC 14-0131 have shorter conidia and wider ascospores.

### Colletotrichum fructicola

Taxon classificationFungiGlomerellalesGlomerellaceae

Prihastuti, L. Cai & K.D. Hyde

Figure 8

#### Description.

Asexual morph formed on CMA. Vegetative hyphae 2.6–5 µm diam (N=20), smooth-walled, septate, branched, hyaline. Appressoria and chlamydospores not observed. Conidiomata 500 × 400 µm (N=3), clustered, sub-globose, smooth-walled, orange. Conidiophores rare, septate, hyaline. Conidia (12.8–) 13.8–16.6 (–18.6) × (2.7–) 3.5–7.8 (–16) µm (N=21), L/W = 2.9, ellipsoidal, smooth-walled, septate, hyaline.

Sexual morph forming on CMA. Ascomata globose, pale brown to dark brown. Peridium (131.9–) 138.4–163.6 (–171.5) × (120.9–) 123.6–142.1 (–143.2) µm (N=4), composed of medium brown, flattened, angular cells. Setae (53–) 57.2–73.1 (–83.3) × (3.4–) 3.5–4(–4.1) µm (N=6), grow on the fruiting body, 2-septate, smooth-walled, contracted at the base, apex slightly rounded, brown to dark brown. Asci (57.6–) 61.2–82.6 (–94.3) × (8.7–) 9.3–13.3 (–15.8) µm (N=12), cylindrical, unitunicate, 8–spored. Ascospores (10–) 12–20 (–20.9) × (3.6–) 4.1–5.2 (–5.3) µm (N=10), L/W = 3.4, ellipsoidal to reniform, somewhat fusiform or acute both ends, 1 to 4 guttules, uni-to bi-seriate, smooth-walled, hyaline.

Cultures on CMA flat with slight serrated margin. Growth rate: 0.9cm/day, with 14-days for sporulation. Cottony, light brown to white from middle to the margin, reverse white to light brown with black spots. Ascomata gregarious and/or isolated. Acervuli and ascomata sometimes gregarious.

**Figure 8.**
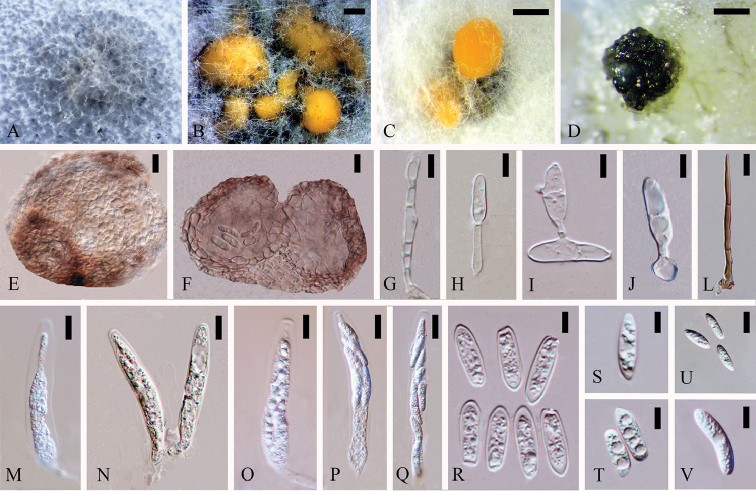
*Colletotrichumfructicola.***A** Colony **B** Conidiomata and ascomata **C, D** Conidiomata **E, F** Ascomata **G–J** Conidiophores **L** Setae **M–Q** Asci **R–V** Ascospores Scale bars: 500 µm (**B–D**), 20 µm (**E, F**), 5 µm (**G–J**), 10 µm (**L**), 10 µm (**M–Q**), 5 µm (**R–V**).

#### Notes.

Strains MFLUCC 14-0087, MFLUCC 14-0148 and MFLUCC 15-0262 had the identical sequences to *Colletotrichumfructicola*. The ITS and GAPDH sequences of them 100% match with many different unpublished species. Blastn researches with ACT sequence of them results in 99% identity with the ex-holotype culture of *C.fructicola* strain ICMP 18581 (GenBank JX009501) isolated from *Coffeaarabica* in Thailand (Weir et al. 2012), which we involved it in phylogenetic analysis. TUB2 sequences of them are 99% identity with *C.boninense* strain CBS 125502 (GenBank KJ955336) as mentioned above. Their ascomata, conidia, asci and ascospores were also similar. Conidia were the same size as the ex-type strain of the pathogen *Colletotrichumfructicola* (9.7–14 × 3–4.3µm) found in coffee berries (Prihastuti et al. 2009). However, ascomata were much smaller and asci as well as ascospores were much larger than the ex-type from coffee berries. In the protologue, *C.fructicola* was introduced with ascomata as 345.67 ± 36.83 × 431.33 ± 69.89 μm, asci as 41.22 ± 7.02 × 7.61 ± 0.58 μm and ascospores as 11.91 ± 1.38 × 3.32 ± 0.35 μm. Here we name strains MFLUCC 14-0087, MFLUCC 14-0148 and MFLUCC 15-0262 isolated from leaves of *Dendrobium* sp. 1 and *Dendrobium* sp. 3, root of *Dendrobium* sp. 2 as *Colletotrichumfructicola*.

### Colletotrichum jiangxiense

Taxon classificationFungiGlomerellalesGlomerellaceae

F. Liu & L. Cai

Figure 9

#### Description.

*Sexual morph* not observed.

Sexual morph not observed. Asexual morph on PDA. Vegetative hyphae 1.3–2.1 µm diam (N=20), smooth-walled, septate, branched, hyaline. Setae and chlamydospores not observed. Conidiophores (12.7–) 13.5–21.4 (–23.4) × (1.9–) 2–3 (–3.2) µm (N=8), branched, hyaline. Conidia (8.6–) 9–12.4 (–13.2) × (3.5–) 3.6–4.4 (–4.5) µm (N=4), L/W = 2.6, ellipsoidal to cylindrical, smooth-walled, aseptate, one end more blunt than the other end, hyaline.

Cultures on PDA flat with entire margin. Growth rate: 0.4cm/day, with 18-days for sporulation. Aerial mycelia dense, cottony, pale to light brown, with brown outline ring close to the edge, mycelia in the middle dark brown, reverse white to light brown.

**Figure 9.**
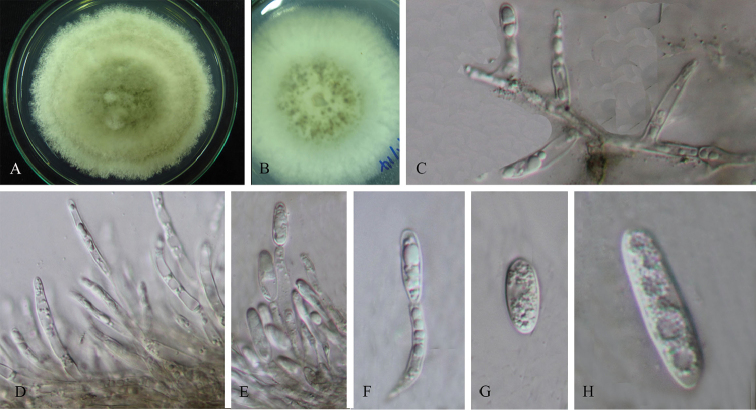
*Colletotrichumjiangxiense.***A** Colony **B** Colony from below **C–F** Conidiophores **G–H** Conidia. Scale bars: 5 µm (**C–F**), 2.5 µm (**G–H**).

#### Notes.

Strains MFLUCC 14-0091 and MFLUCC 14-0092 were the same species as they grouped with high support (98ML/1.0BI/87MP). They formed a very close clade with the pathogen *C.jiangxiense* isolated from *Camellia.* However, different media were used in these two studies. Blastn researches with ITS sequences results in 100% identity with *C.gloeosporioides* strain SS1-MS1 (GenBank KP900279) isolated from *Huperziaserrate* in China (Wang et al. 2016). GAPDH, ACT and TUB2 sequences of MFLUCC 14-0091 and MFLUCC 14-0092 are closest to C.kahawaesubsp.ciggaro strain ICMP 18534 (GenBank JX009904) with 98% identity isolated from *Kunzeaericoides* in New Zealand, 99% identity with strain ICMP 12952 (GenBank JX009431) isolated from *Persea Americana* in New Zealand, and 99% identity with strain CO22-1 (GenBank KJ001124) isolated from *Rubusglaucus* in Colombia respectively (Weir et al. 2012; Afanador-Kafuri et al. 2014). Conidia size reported for *C.jiangxiense* was 15.2 ± 1 × 5.2 ± 0.4 μm, which was larger and faster growing than the strains isolated in this study. There were 5bp differences between strain MFLUCC 14-0091 and *C.jiangxiense*. Here we name both of isolates from leaves of *Dendrobium* sp. 1 as *C.jiangxiense*.

### Colletotrichum orchidophilum

Taxon classificationFungiGlomerellalesGlomerellaceae

Damm, P.F. Cannon & Crous

Figure 10

#### Description.

*Sexual morph* not observed.

Sexual morph not observed. Asexual morph on SDA. Vegetative hyphae 1.9–5.4 µm diam, smooth-walled, septate, branched, hyaline to pale brown. Chlamydospores not observed. Appressoria brown, smooth-walled. Conidiomata superficial or under mycelia, smooth-walled, 200 × 300 µm, black. Conidiophores smooth-walled, branched or unbranched, hyaline. Conidiophores and appressoria rare. Conidia (9.6–) 11.7–14.1 (–14.7) × (2.9–) 3.5–4.4 (–4.8) µm, L/W = 3.3, cylindrical, straight, with 1 to 4 guttules, one end somewhat acute, hyaline.

Cultures on SDA flat with entire margin. Growth rate: 0.44cm/day, with nearly 20-days for sporulation. White with dark green mycelia around the middle, white edge, reverse white. Cultures on PDA flat with entire margin. Growth rate: 0.45cm/day, with 30-days for sporulation. Fluffy, white, reverse light brown. Acervuli in mass black, irregular, asymmetrical, merging in media.

**Figure 10.**
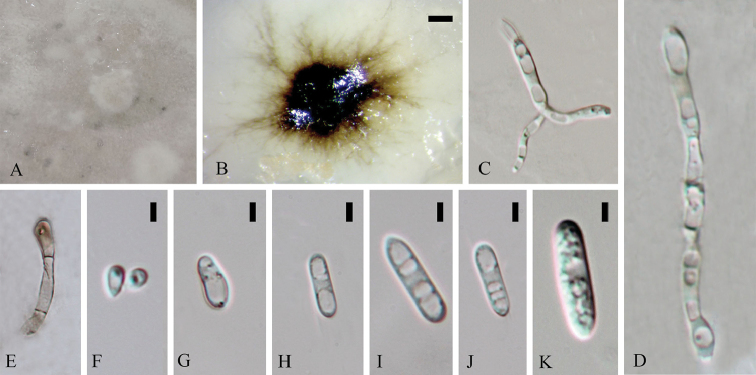
*Colletotrichumorchidophilum.***A** Colony **B** Fruiting body **C–D** Conidiophores **E** Appressoria **F–K** Conidia. Scale bars: 200 µm (**B**), 5 µm (**F–K**).

#### Notes.

Strains MFLUCC–14–0161 and MFLUCC–14–0162 belong to a single species as they have similar conidia, cultures and the nearly identical sequence data. The support values of 100/1.00/100 totally grouped them with *C.orchidophilum* and their branch lengths are slightly different. Blastn researches sequences of MFLUCC 14-0161 and MFLUCC 14-0162 results in 99% identity with ITS (GenBank NR111729), GAPDH (GenBank JQ948481) and ACT (GenBank JQ949472) sequences of ex-holotype culture of *C.orchidophilum* strain CBS 632.80 isolated form *Dendrobium* sp. in USA (Damm et al. 2012). TUB2 sequence is 99% identity with pathogenic *C.fructicola* strain AV24 (GenBank KX786459) isolated from grapevine shoots in Brazil (Santos et al. 2018) and *C.gloeosporioides* strain TL-2 (GenBank KC913205) isolated from *Camelliasinensis* in China (Guo et al. 2014). Because no conidiophores were detected in culture, no measurement for the conidiophores could be given. In this study, strains MFLUCC 14-0161 and MFLUCC 14-0162 of *C.orchidophilum* were isolated from leaves of *D.harveyanum*.

### Colletotrichum boninense

Taxon classificationFungiGlomerellalesGlomerellaceae

Moriwaki, Toy. Sato & Tsukib.

For an illustrated description please refer Damm et al. (2012a).

#### Notes.

Strains MFLUCC 14-0086, MFLUCC 14-0124 and MFLUCC 15-0261 grouped with *C.boninense* and MFLUCC 14-0128. All have very similar sequences as those as the ex-type of with *C.boninense* (only 2bp difference), while there was 11 base pair deviations between these strains and *Colletotrichumdoitungense* sp. nov. Blastn researches with ITS sequences of them result in 100% identity with ITS sequence of endophytic *C.boninense* strain SL-ML18 (GenBank KP900269) isolated from *Huperziaserrate* in China (Wang et al. 2016) and strain CGMCC 3.15168 (GenBank KC244158) as mentioned above. GAPDH and ACT sequences of them are 97% identity with *C.boninense*CGMCC 3.15168 (GenBank KC843491) and 100% identity with *C.fructicola* strain 1104-7 (GenBank KX885159) isoalted from *Malusdomestica* in China (Liang et al. 2017). TUB2 blastn result are 99% identity with *C.fructicola* strain AV24 (GenBank KX786459) and *C.gloeosporioides* strain TL-2 (GenBank KC913205) as mentioned above. Here we identify these three strains isolated from leaves of *D.catenatum* and *Dendrobium* sp. 1, stem of *D.* sp. 2 respectively as *Colletotrichumboninense*.

### Colletotrichum

Taxon classificationFungiGlomerellalesGlomerellaceae

sp. indet

#### Notes.

Strain MFLUCC 14-0120 failed to sporulate and lacks a complete morphological description. It formed a single branch close to *C.camelliae-japonicae*, MFLUCC 14-0129 / MFLUCC 14-0131 with 67ML/1.00BI/62MP support. There were 15bp and 11bp differences mainly in the ACT gene region among MFLUCC 14-0120 and *C.camelliae-japonicae*, MFLUCC 14-0129/MFLUCC 14-0131 respectively. ITS sequence blastn of MFLUCC 14-0120 showed many different kinds of species with 99% identity. Blastn searches with GAPDH (GenBank KC293736) and TUB2 (GenBank KC293656) sequences result in 99% identity with *C.citricola* strain SCX 151 as mentioned above. The ACT of MFLUCC–14–0120 is 98% identity with *C.boninense* strain CBS 125502 (GenBank KJ954462) as mentioned above. Here we listed it as an unidentified species.

## Discussion

### *Colletotrichum* species associated with orchid species

Many *Colletotrichum* species have been isolated from Orchidaceae plants sampled in China in previous studies (e.g. Yang et al. 2011; Chen et al. 2012; Tao et al. 2008, 2013). Eighteen *Colletotrichum* species have been reported from these studies. For example, *Colletotrichumbeeveri* isolated from *Pleionebulbocodioides*; *C.bletillum* and *C.caudasporum* isolated from *Bletillaochracea*; *C.oncidii* isolated from *Oncidium* sp. (Yang et al. 2011; Damm et al. 2012a; Tao et al. 2013). The present study is the first to report endophytic fungi from *Dendrobium* spp. in Thailand combining both multi-loci sequence data and morphological characteristics. *Colletotrichum* species in this study were diverse and present in every *Dendrobium* sample collected from all sites. Therefore, we conclude that *Orchidaceae* plants are rich source of endophytic *Colletotrichum* species.

### Methods affecting the identification

Hyde and Zhang (2008) and Hyde et al. (2009b) suggested that nucleotide sequence data of holotypes or epitypes is essential for analysing phylogenetic relationships among *Colletotrichum* species. A polyphasic method combining morphological characteristics and molecular phylogenetics has been applied to define and re-order species in this genus (Cai et al. 2009; Hyde et al. 2009; Damm et al. 2012a, b, c; Jayawardena et al. 2016a, b).

We found some differences in the *Colletotrichumgloeosporioides* species complex backbone tree as compared to that constructed with more genes in Weir et al (2012), Udayanga et al (2013) and Jayawardena et al. (2016a). *Colletotrichumjiangxiense* clusters with *C.rhexiae* rather than *C.kahawae*. *C.fructicola* is closer to *C.siamense* rather than *C.nupharicola*. The genes CHS-1 and HIS3 were not involved in this study and may be responsible for the differences. Actually CHS-1 and HIS3 could resolve species in sevaral other species complexes of *Colletotrichum* (Jayawardena et al. 2016a). However, the combination of ApMat and GS turned out to be the most effective genes in species resolution in the *Colletotrichumgloeosporioides* species complex (Liu et al. 2015). Our study is the first to use multiple gene sequences to analyse fungal endophytes from *Dendrobium* orchids.

### Relationship between *Colletotrichum* and *Dendrobium*

Few species identified in this study showed host-specificity. Nevertheless, this study provides evidence that *C.orchidophilum* colonizes a wide range of hosts in *Orchidaceae* (Damm et al. 2012b). In addition, we found that leaves contained higher numbers of *Colletotrichum* species (11 strains from leaves) than other parts (4 strains from roots and 7 strains from stems). All *Dendrobium* leaves in this study were colonized by *Colletotrichum* strains. Our results are similar to those of Chen et al. (2011) who isolated more *Colletotrichum* species from stems and leaves of *Dendrobium* species than that from roots.

The majority of *Colletotrichum* species isolated from *Dendrobium* species in this study were fungal endophytes. This was also reported by Chen et al. (2011) and Yuan et al. (2009). The most common fungal endophytes in leaves of *Lepanthesrupestris* (*Orchidaceae*) sampled in a Puerto Rican forest were a *Colletotrichum* species which showed antagonism against other fungal taxa (Bayman et al. 2002). Most *Colletotrichum* species have been identified as plant pathogens living a hemibiotrophic life strategy, they adopt a biotrophic phase at an early stage and switch to a necrotrophic phase later (Damm et al. 2010; Cannon et al. 2012).

Here we speculate that most isolates in this study might be latent pathogens (Photita et al. 2004), since in the phylogenies, they were nested with pathogenic strains or have previously been reported to cause plant diseases (Tao et al. 2013, Hou et al 2016). *Colletotrichumjiangxiense* was isolated as a pathogen from leaf lesions of *Camellia* sp. (Liu et al. 2015). *Colletotrichumboninense* was reported as an anthracnose causing agent from *Dendrobiumkingianum* in Japan (Moriwaki et al. 2003).

## Supplementary Material

XML Treatment for Colletotrichum cariniferi

XML Treatment for Colletotrichum chiangraiense

XML Treatment for Colletotrichum watphraense

XML Treatment for Colletotrichum doitungense

XML Treatment for Colletotrichum parallelophorum

XML Treatment for Colletotrichum citricola

XML Treatment for Colletotrichum fructicola

XML Treatment for Colletotrichum jiangxiense

XML Treatment for Colletotrichum orchidophilum

XML Treatment for Colletotrichum boninense

XML Treatment for Colletotrichum

## Appendix A

**Table:** Fungal isolates and sequences of region/genes used in *Colletotrichum* phylogenetic analysis.

Species	Isolate^a^	GenBank accession number			
		ITS	GAPDH	ACT	ß–tubulin
* C. acutatum *	CBS 128531*	JQ005776	JQ948677	JQ005839	JQ005860
* C. aeschynomenes *	CBS 128532*	JX010176	JX009930	JX009483	JX010392
* C. alcornii *	CBS 128534*	JX076858	–	–	–
* C. alienum *	ICMP 12071*	JX010251	JX010028	JX009572	JX010411
* C. annellatum *	CBS 128536*	JQ005222	JQ005309	JQ005570	JQ005656
* C. anthrisci *	CBS 125334*	GU227845	GU228237	GU227943	GU228139
* C. aotearoa *	CBS 128538*	JX010205	JX010005	JX009564	JX010420
* C. arxii *	CBS 132511*	NR132055	KF687843	KF687802	KF687881
* C. australe *	CBS 128540*	JQ948455	JQ948786	JQ949776	JQ950106
* C. beeveri *	CBS 128541*	JQ005171	JQ005258	JQ005519	JQ005605
* C. bidentis *	CBS 128542*	KF178481	KF178506	KF178578	KF178602
* C. bletillum *	CBS 128543*	JX625178	KC843506	KC843542	JX625207
* C. boninense *	CBS 123755*	JQ005153	JQ005240	JQ005501	JQ005588
* C. brasiliense *	CBS 128545*	JQ005235	JQ005322	JQ005583	JQ005669
* C. brassicola *	CBS 128546*	JQ005172	JQ005259	JQ005520	JQ005606
* C. brevisporum *	CBS 128547*	JQ247623	JQ247599	JQ247647	JQ247635
* C. camelliae *	ICMP 10643	JX010224	JX009908	JX009540	JX010436
* C. camelliae-japonicae *	CGMCC3.18117*	KX853165	KX893583	KX893575	KX893579
* C. caudasporum *	CGMCC 3.15106*	JX625162	KC843512	KC843526	JX625190
* C. cereale *	CBS 129663	JQ005774	–	JQ005837	JQ005858
* C. chlorophyti *	IMI 103806*	GU227894	GU228286	GU227992	GU228188
* C. chrysanthemi *	IMI 364540	JQ948273	JQ948603	JQ949594	JQ949924
* C. citricola *	SXC 151*	KC293576	KC293736	KC293616	KC293656
* C. clidemiae *	ICMP 18658*	JX010265	JX009989	JX009537	JX010438
* C. cliviae *	CBS 125375*	JX519223	GQ856756	JX519240	JX519249
* C. coccodes *	CBS 369.75	JQ005775	HM171673	JQ005838	JQ005859
* C. colombiense *	CBS 129818*	JQ005174	JQ005261	JQ005522	JQ005608
* C. cordylinicola *	ICMP 18579*	JX010226	JX009975	HM470234	JX010440
* C. curcumae *	IMI 288937*	GU227893	GU228285	GU227991	GU228187
* C. cymbidiicola *	IMI 347923*	JQ005166	JQ005253	JQ005514	JQ005600
* C. dematium *	CBS 125.25*	GU227819	GU228211	GU227917	GU228113
* C. dracaenophilum *	CBS 118199*	JX519222	–	JX519238	JX519247
* C. echinochloae *	MAFF 511473*	AB439811	–	–	–
* C. eleusines *	MAFF 511155*	JX519218	–	JX519234	JX519243
* C. endophytum *	CGMCC 3.15108*	JX625177	KC843521	KC843533	JX625206
* C. eremochloae *	CBS 129661*	CBS 129661	–	JX519236	JX519245
* C. excelsum-altitudum *	CGMCC 3.15130*	HM751815	KC843502	KC843548	JX625211
* C. falcatum *	CBS 147945*	JQ005772	–	JQ005835	JQ005856
* C. fioriniae *	CBS 128517*	JQ948292	JQ948622	JQ949613	JQ949943
* C. fructi *	CBS 346.37*	GU227844	GU228236	GU227942	GU228138
* C. fructicola *	ICMP 18581*	JX010165	JX010033	FJ907426	JX010405
* C. fructivorum *	Coll1414*	JX145145	–	–	JX145196
* C. fusiforme *	MFLU 130291*	NR138010	KT290255	KT290251	KT290256
* C. gigasporum *	MUCL 44947*	AM982797	–	–	FN557442
* C. godetiae *	CBS 133.44*	JQ948402	JQ948733	JQ949723	JQ950053
* C. graminicola *	CBS 130836*	JQ005767		JQ005830	JQ005851
* C. grevilleae *	CBS 132879*	KC297078	KC297010	KC296941	KC297102
* C. guizhouensis *	CGMCC 3.15112*	JX625158	KC843507	KC843536	JX625185
* C. hanaui *	MAFF 305404*	JX519217	–	–	JX519242
* C. henanense *	LF238*	KJ955109	KJ954810	–	KJ955257
* C. hippeastri *	CBS 125376*	JQ005231	JQ005318	JQ005579	JQ005665
* C. hemerocallidis *	CDLG5*	JQ400005	JQ400012	JQ399991	JQ400019
* C. horii *	ICMP 10492	GQ329690	GQ329681	JX009438	JX010450
* C. incanum *	ATCC 64682*	KC110789	KC110807	KC110825	KC110816
* C. jasminigenum *	MFU 10–0273*	HM131513	HM131499	HM131508	HM153770
* C. jiangxiense *	LF 488*	KJ955149	KJ954850	KJ954427	–
* C. kahawae *	ICMP 17816*	JX010231	JX010012	JX009452	JX010444
* C. kartsii *	CORCG 6*	HM585409	HM585391	HM581995	HM585428
* C. laticiphilum *	CBS 112989*	JQ948289	JQ948619	JQ949610	JQ949940
* C. lilii *	CBS 109214	GU227810	GU228202	GU227908	GU228104
* C. lindemuthianum *	CBS 144.31*	JQ005779	JX546712	JQ005842	JQ005863
* C. linicola *	CBS 172.51	JQ005765	–	JQ949476	JQ949806
* C. liriopes *	CBS 119444*	GU227804	GU228196	GU227902	GU228098
*C.magnisporu*m	CBS 398.84	KF687718	KF687842	KF687803	KF687882
* C. malvarum *	CBS 527.97*	KF178480	KF178504	KF178577	KF178601
* C. menispermi *	MFLU 14–0625*	KU242357	KU242356	KU242353	KU242354
* C. miscanthi *	MAFF 510857*	JX519221	–	JX519237	JX519246
* C. musae *	ICMP 19119*	JX010146	JX010050	JX009433	HQ596280
* C. navitas *	CBS 125086*	JQ005769	–	JQ005832	JQ005853
* C. nicholsonii *	MAFF 511115*	JQ005770	–	JQ005833	JQ005854
* C. novae-zelandiae *	CBS 128505*	JQ005228	JQ005315	JQ005576	JQ005662
* C. nupharicola *	ICMP 18187*	JX010187	JX009972	JX009437	JX010398
* C. ochraceae *	CGMCC 3.15104*	JX625156	KC843513	KC843527	JX625183
* C. oncidii *	CBS 129828*	JQ005169	JQ005256	JQ005517	JQ005603
* C. orchidophilum *	CBS 632.80*	JQ948151	JQ948481	JQ949472	JQ949802
* C. parsonsiae *	CBS 128525*	JQ005233	JQ005320	JQ005581	JQ005667
* C. paspali *	MAFF 305403*	JX519219	–	JX519235	JX519244
* C. petchii *	CBS 378.94*	JQ005223	JQ005310	JQ005571	JQ005657
* C. phaseolorum *	CBS 157.36	GU227896	GU228288	GU227994	GU228190
* C. phyllanthi *	CBS 175.67*	JQ005221	JQ005308	JQ005569	JQ005655
* C. pseudoacutatum *	CBS 436.77*	JQ948480	JQ948811	JQ949801	JQ950131
* C. pseudomajus *	CBS 571.88*	NR132059	KF687826	KF687801	KF687883
* C. psidii *	ICMP 19120	JX010219	JX009967	JX009515	JX010443
* C. radicis *	CBS 529.93*	NR132057	KF687825	KF687785	KF687869
* C. rhexiae *	Coll 1026*	JX145128	–	–	JX145179
* C. rhombiforme *	CBS 129953*	JQ948457	JQ948788	JQ949778	JQ950108
* C. riograndense *	COAD 928*	KM655299	KM655298	KM655295	KM655300
* C. rusci *	CBS 119206*	GU227818	GU228210	GU227916	GU228112
* C. salsolae *	ICMP 19051*	JX010242	JX009916	JX009562	JX010403
* C. siamense *	ICMP 18578*	JX010171	JX009924	FJ907423	JX010404
* C. sichuanensis *	LJTJ3	KP748193	KP823773	KP823738	KP823850
* C. spinaciae *	CBS 128.57	GU227847	GU228239	GU227945	GU228141
* C. sublineola *	CBS 131301*	JQ005771	–	JQ005835	JQ005855
* C. syzygicola *	DNCL 021*	KF242094	KF242156	KF157801	KF254880
* C. tanaceti *	CBS 132693*	–	JX218243	JX218238	JX218233
* C. tebeestii *	CBS 522.97*	KF178473	KF178505	KF178570	KF178594
* C. temperatum *	Coll 883*	JX145159	–	–	JX145211
* C. theobromicola *	ICMP 18649	JX010294	JX010006	JX009444	JX010447
* C. ti *	ICMP 4832*	JX010269	JX009952	JX009520	JX010442
* C. torulosum *	CBS 128544*	JQ005164	JQ005251	JQ005512	JQ005598
* C. trichellum *	CBS 217.64*	GU227812	GU228204	GU227910	GU228106
* C. trifolii *	CBS 158.83*	KF178478	KF178502	KF178575	KF178599
* C. tropicicola *	BCC 38877*	JN050240	JN050229	JN050218	JN050246
* C. trucatum *	CBS 151.35	GU227862	GU228254	GU227960	GU228156
* C. verruculosm *	IMI 45525*	GU227806	GU228198	GU227904	GU228100
* C. vietnamense *	CBS 125478*	KF687721	KF687832	KF687792	KF687877
* C. viniferum *	GZAAS 5.0860*1**	JN412804	JN412798	JN412795	JN412813
*C.$1×anthorrhoeae*	ICMP 17903*	JX010261	JX009927	JX009478	JX010448
* C. yunnanense *	CGMCC AS3.9167*	EF369490	–	JX519239	JX519248
* Australiasca queenslandica *	BRIP 24607	HM237327	–	–	–
* Monilochaetes guadalcanalensis *	CBS 346.76	GU180625	–	–	–
* Monilochaetes infuscans *	CBS 869.96	JQ005780	JX546612	JQ005843	JQ005864

^a^Isolates marked with “*” are extype or ex-epitype strains.

## Appendix B

**Table:** Fungal isolates and sequences of region/genes in this study.

Species	Isolate	GenBank accession number			
		ITS	GAPDH	ACT	ß–tubulin
* C. boninense *	MFLU 14-0124	MG792809	MK165700	–	MH351286
	MFLU 14-0086	MG792816	MH673668	MH376390	MH351281
	MFLU 14-0261	MG792815	MK165703	MH376400	MH351292
* C. cariniferi *	MFLU 14-0100	MF448521	–	–	MH351274
* C. chiangraiense *	MFLU 14-0119	MF448522	–	MH376383	MH351275
* C. citricola *	MFLU 14-0129	MG792821	MK165697	MH376395	MH351287
	MFLU 14-0131	MG792822	MK165696	MH376396	MH351288
* C. doitungense *	MFLU 14-0128	MF448524	MH049480	MH376385	MH351277
* C. fructicola *	MFLU 14-0087	MG792812	MK165691	MH376391	MH351282
	MFLU 14-0262	MG792814	MK165698	MH376401	MH351293
	MFLU 14-0148	MG792813	MK165701	MH376397	MH351289
* C. jiangxiense *	MFLU 14-0091	MG792806	MH673669	MH376392	MH351283
	MFLU 14-0092	MG792807	MH673670	MH376393	MH351284
* C. orchidophilum *	MFLU 14-0161	MG792818	MK165702	MH376398	MH351290
	MFLU 14-0162	MG792819	MK165704	MH376399	MH351291
* C. parallelophorum *	MFLU 14-0085	MF448525	MK165695	–	MH351280
	MFLU 14-0077	MG792808	MK165692	MH376387	MH351279
	MFLU 14-0079	MG792820	MK165693	MH376388	–
	MFLU 14-0082	MG792810	MK165694	MH376389	–
	MFLU 14-0083	MG792811	MH049478	MH376386	MH351278
*C.* sp. indet.	MFLU 14-0120	MG792817	MK165699	MH376394	MH351285
* C. watphraense *	MFLU 14-0123	MF448523	MH049479	MH376384	MH351276
